# Tracking contaminants of concern in wet-weather sanitary sewer overflows

**DOI:** 10.1007/s11356-023-29152-x

**Published:** 2023-08-15

**Authors:** Colin H. Besley, Graeme E. Batley, Michele Cassidy

**Affiliations:** 1 Laboratory Services, Sydney Water, 51 Hermitage Road, West Ryde, NSW 2114 Australia; 2 CSIRO Environment, Locked Bag 2007, Kirrawee, NSW 2232 Australia; 3 Wastewater Product, Sydney Water, 1 Smith Street, Parramatta, NSW 2150 Australia

**Keywords:** Organic chemicals, Influent, Sanitary sewage, Autosamplers, Ammonia, Dilution

## Abstract

Four representative sites in the greater city of Sydney, Australia, were selected for a study of the wet-weather overflow of sanitary (separate to stormwater) sewerage systems. Water samples were collected by autosamplers from up to eight wet weather overflow events over 16 months and from companion receiving water sites. The objective was to identify the risks posed by sewage contaminants to aquatic biota in the receiving waters, to aid in prioritising management actions. Twelve organic contaminants were identified in influents across the four sites under rainfall ingress diluted conditions, with measurements showing that the highest concentrations were restricted to the anti-inflammatory acetaminophen and the diabetes medication metformin. Lesser contaminants included theobromine, ibuprofen, sucralose, and three benzotriazoles (mainly 1-H benzotriazole). An assessment of the toxicity of the identified organic chemicals indicated that none appeared to pose concerns for ecosystem health before wet-weather ingress dilution, and this was even less likely after dilution in the receiving waters. Metal concentrations were low; however, ammonia concentrations in the influent did pose a risk to ecosystem health, although receiving water dilution diminished this risk at four of the five receiving water locations studied.

**Supplementary Information:**

The online version contains supplementary material available at 10.1007/s11356-023-29152-x.

## Introduction

A common problem for water authorities in large urban mega-cities is the pressure that urban population growth is placing on wastewater management. Sydney (Australia) is facing such problems. With a metropolitan population that has grown from 2.1 million in 1960 to 5.1 million in 2022, its sewerage system managed by Sydney Water needs continuous improvement to cope with the increasing discharges and the impacts of rainfall, especially in La Niña climate years, that is resulting in sanitary sewer overflows. With over 3000 emergency relief structures (ERSs, designed overflow points) on trunk and reticulation pipes in its sewerage system that extends some 27,000 km, they are undertaking a comprehensive risk assessment to prioritise those areas under greatest stress for improved management. The ERSs are designed and positioned along the sewerage system to allow wet weather overflows to escape to receiving waters rather than overflow onto properties. Overflows occur through the entry of stormwater through incorrect stormwater connections, and when ingress and inflow through cracks or faults in sewer pipes exceeds the hydraulic capacity of the sewerage system. Hydraulic capacity is typically three times the average dry weather flow volume.

A key component of this exercise is the identification of contaminants of concern in the overflows and evaluation of their potential to adversely impact biota. Ammonia has been previously identified together with chlorine as the most important toxicants immediately downstream of effluent (treated sewage) discharges (Davis 1997). Environment Canada (2001) also noted that unionised ammonia was the most frequent cause of toxicity from wastewater effluent. Camargo and Alonso (2006) suggested ammonia, nitrite, and nitrate can contribute to direct toxicity of aquatic organisms, and Quijano et al. (2017) stated combined sewer overflows are a major source of carbonaceous biochemical oxygen demand and ammonia. Metals in influent are also contaminants of concern. Drozdova et al. (2015) suggested that the occurrence of metals in influent depends on the number of connected inhabitants and on the human activities from industries and households. A literature review of sources of metal contaminants in domestic wastewater from household studies in Australia indicated that major inputs were from the metals Pb, Zn, and Cu, with As, Ni, and Hg near detection limits. Inputs of lead appeared to originate from the laundry and bathroom, while zinc mainly originated from the bathroom, and the major sources of copper were from plumbing and water supply (Tjadraatmadja and Diaper 2006).

A recent study by Allinson et al. (2023) used passive samplers to identify some 254 organic chemicals present in receiving waters with inflows from both sanitary sewer overflows and urban stormwater. The technique does not easily translate passive sampler detection to receiving water concentrations. The current study reports a more focussed investigation of chemicals collected by autosamplers triggered by overflow events. Attempts have then been made to assess the ecological risk based on published toxicity data. Further studies are in progress using whole effluent toxicity testing to determine safe dilutions of identified contaminants of concern and to determine whether these are achieved.

Launay et al. (2016) noted that combined sewer overflows (CSO) represented an important pathway for a wide range of contaminants from wastewater systems to enter urban receiving waters. Fent et al. (2006) indicated that a better evaluation of the concentrations of contaminants in CSO was needed to assess the ecological risk from these contaminants in receiving waters. Gromaire et al. (2001) suggested that concentrations of contaminants in CSO were influenced by the dilution of sewage by stormwater, by an internal contribution of in-sewer sediment resuspension, and by external runoff. In the current study, stormwater ingress into the sanitary sewerage system was the main driver of sanitary sewer overflows as the stormwater system in Sydney is separated from the sewerage system. To the best of our knowledge, no other studies have assessed the ecological risk of contaminant concentrations from sanitary sewer overflows by tracking a suite of contaminants detected in influent and in receiving water concentrations across multiple sites.

The current study was conducted to assess 18 detected organic contaminants (Table 1) together with ammonia and metals in the sanitary sewer system. The study had four objectives: (i) to examine the types of contaminants detected in the water column of the sewer and in associated downstream receiving waters; (ii) to review the aquatic toxicity of water-column-detected contaminants; (iii) to determine if the water-column-detected organic contaminants were similar across the four sewer carriers of the sanitary sewer system and associated five receiving water sites; and (iv) to assess dilution of influent in the receiving waters. An additional line of evidence to assess dilution was provided by companion human-associated microbial source tracking (MST) marker-gene data.

**Table 1:** Suppliers of standard compounds and limits of quantitation for sewer overflow chemicals

Chemical	Supplier and compound number	Limit of quantitation (μg/L)
Acetaminophen	Aldrich or A-064 Cerilliant	1.0
Atenolol	Sigma # A7655-1G or A-072 Cerilliant	1.0
Benzophenone-3 (BP3)	ALR-081S-CN - ACCUSTANDARD	1.0
1H-benzotriazole	Sigma # 76457-50mg	0.25
Cotinine	C-016-1ML Cerilliant	1.0
Diclofenac	LGC Dr. Ehrenstorfer # DRE-A12537000AL-100	1.0
Disodium distyrylbiphenyl disulfonate (FB351)	TRC-B535200 Toronto Research Chemicals	1.0
Hydrochlorothiazide	H-001-1ML Cerilliant	1.0
Ibuprofen	Sigma #17905-1G or I-009 Cerilliant	1.0
Metformin	M-072-1ML Cerilliant	1.0
4-Methyl-1H-benzotriazole	Sigma # 14593-50mg	0.25
5-Methyl-1H-benzotriazole	Sigma# 14949-50mg	0.25
Naproxen	Fluka #46482 or N-042 Cerilliant	1.0
Sotalol	LGC Dr. Ehrenstorfer # DRE-A16972630AL-100	1.0
Sucralose	LGC Dr. Ehrenstorfer # DRE-C16985800	1.0
Sulfapyridine	LGC Dr. Ehrenstorfer # DRE-A17000100AL-1000	1.0
Theobromine	T-016-1ML Cerilliant	1.0
p-Toluenesulfonamide	LGC Dr. Ehrenstorfer # DRE-C17594700	1.0
Ammonia NH_3_–N	AR ammonia	10.0
Total iron	ICPMS standards	5.0
Filterable aluminium	ICPMS standards	5.0
Filterable iron	ICPMS standards	5.0
Filterable copper	ICPMS standards	0.5
Filterable manganese	ICPMS standards	0.5
Filterable lead	ICPMS standards	0.1
Filterable zinc	ICPMS standards	1.0
Filterable cadmium	ICPMS standards	0.1
Filterable selenium	ICPMS standards	0.2

## Materials and methods

### Sampling site selection

**Fig. 1:**
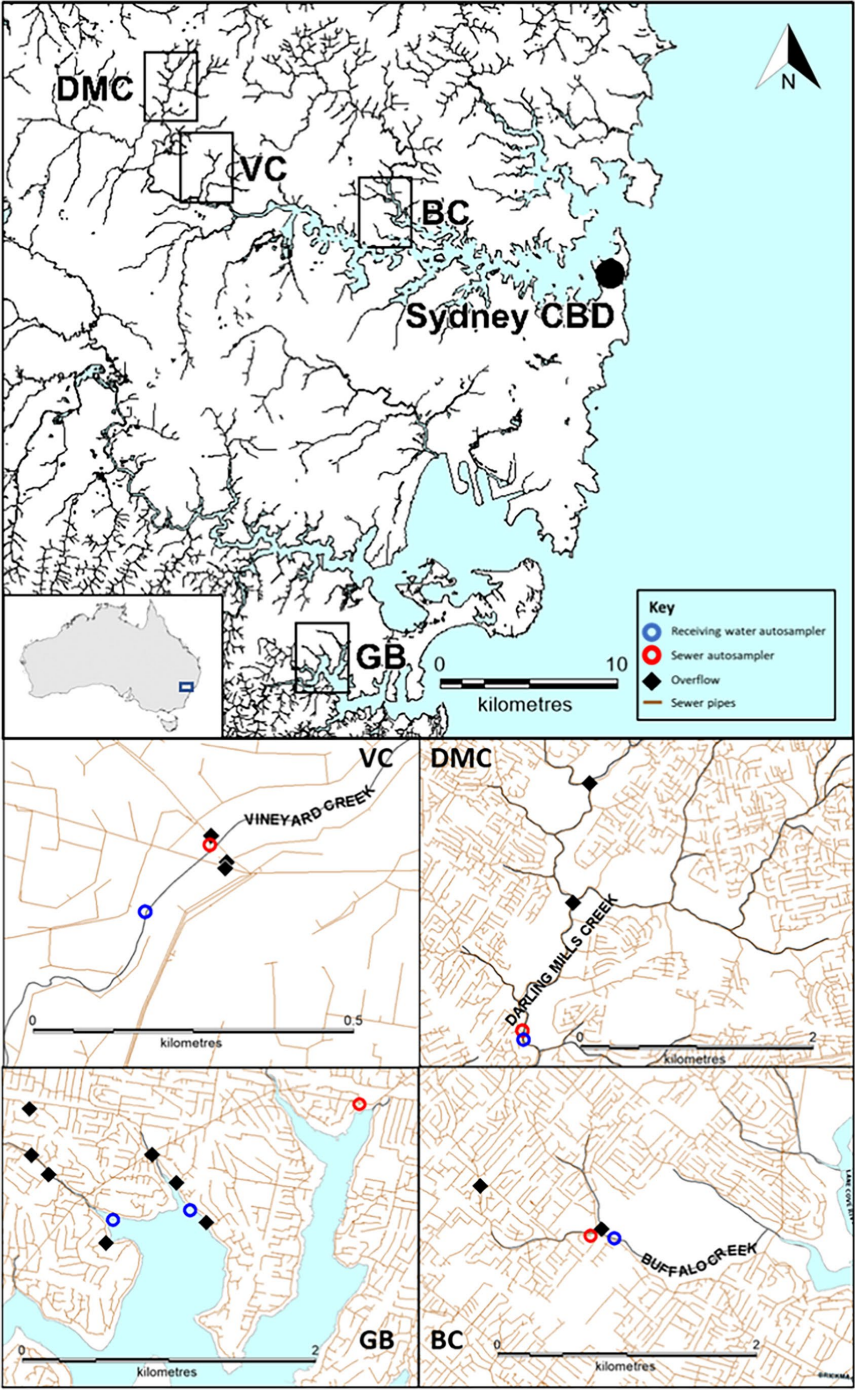
Overall location map and detail of each of the four study sites: VC, Vineyard Creek; DMC, Darling Mills Creek; GB, Gymea Bay; BC, Buffalo Creek. Red circles represent sewer autosamplers, blue circles represent downstream receiving water autosamplers, black diamonds indicate position in sewer catchments of gauged ERS (sewer overflow points), and brown lines represent sewer trunk and reticulation pipes of these urban study locations

The selection of a range of ERS locations was based on modelled overflow volumes from a larger cohort of some 1000 modelled overflow points across the four major sewerage networks of the Sydney metropolitan area. Modelled overflow volumes were obtained from a hydraulic sewer system model developed in response to licence requirements (NSW EPA, 2022). The ERS site selection approach of the current study was also employed for the study of the composition of gross contaminants contained in wet-weather overflows in the megacity of Sydney, Australia (Besley and Cassidy, 2021), along with the consideration of safe access for field staff under various weather conditions. ERSs at four locations in the sanitary sewerage system were selected for study. These overflowed (spilled) to Gymea Bay, Gymea; Darling Mills Creek, Baulkham Hills; Buffalo Creek, East Ryde; and Vineyard Creek, Dundas (Fig. 1). They represented one low, two medium, and one high volume and frequency wet-weather overflows, respectively (Supporting information Table S1).

Darling Mills Creek, Buffalo Creek, and Vineyard Creek are situated northwest of the Sydney central business district (CBD). These three freshwater creeks flow into the upper reaches of Sydney Harbour. The fourth location at Gymea Bay is situated in the Port Hacking estuary, located south-southwest of the Sydney CBD (Fig. 1).

### Field sample collection

Water samples were collected with ISCO Avalanche portable refrigerated (4 °C) autosamplers at the four locations of the sewerage system (Fig. 1). Across these locations, five autosamplers were set up at receiving water sites, comprising two in Gymea Bay and one at each respective downstream ERS site of the three freshwater streams, together with an autosampler setup at each sewer carrier that transited the four locations (Fig. 1). To collect influent water column samples, the autosamplers were triggered by spider-logger telemetry from hydrostatic sensors installed at Buffalo, Vineyard, and Gymea, while a Pulsar dBi6 sensor best accounted for the pipe configuration at Darling Mills. When sensors installed within an ERS measured discharge flows of 20 mm above the overflow weir of the ERS, and sustained above this level for 15 min, a message was relayed to the spider logger that then triggered a start message to the respective autosamplers. Companion SMS text messaging alerted when discharge flows had commenced, and this messaging was used to subsequently deploy field teams to retrieve water samples for laboratory processing. Wet-weather samples were collected from 24 events with four collections from Darling Mills, five from Buffalo, seven from Gymea, and eight from Vineyard between October 2018 and February 2020.

Dry-weather influent samples were collected on two occasions (in June 2019) by manually triggering the autosampler at the Vineyard sewer location.

To assess receiving waters downstream of the study ERSs when wet-weather overflow spills were not gauged to have occurred, the receiving water autosamplers were triggered to start water sample collection off rainfall gauges when a rainfall threshold of 2 mm intensity in 15 min was met. Ten events with four collections from Darling Mills and six events from Vineyard were sampled within October 2018 and February 2020.

The refrigerated autosamplers were triggered to collect discrete samples into 14 carousel 950 mL bottles, filled 5 min apart. Depending on the duration of the ERS spill event, the first five carousel bottles were generally selected for laboratory processing. If a bottle did not fill properly, potentially due to a temporary blockage by a gross pollutant such as a wet wipe, then the next filled bottle was selected provided the gauging records indicated that the ERS spill was still in progress. After mixing by inversion several times, water samples from each selected carousel bottle were transferred to sample containers with unique laboratory traceability numbers. Sample containers comprised 100 mL polyethylene terephthalate (PET) for ammonia testing, 250 mL high-density polyethylene for metals, and 500 mL brown glass bottles for organics testing. These were then placed on ice in an Esky cooler at approximately 4 °C for delivery to the Sydney Water laboratory, which generally occurred within 12 h of initial collection.

Human-associated MST marker genes were assessed from influent collected under diluted rainfall ingress conditions on six occasions between October 2020 and March 2021, comprising one collection each from the sewers of Buffalo and Gymea, and on two occasions each from the Darling Mills and Vineyard sewers. Samples from each event were collected in triplicate, 5 min apart. Dry-weather influent concentrations of human-associated MST marker gene concentrations were also measured from triplicate samples collected at influent sample points of two wastewater treatment plants in the Sydney region on 12 occasions from September 2019 to late September 2020 in a study by Ahmed et al. (2022). They investigated the distribution of wastewater-associated marker genes and their association with pathogenic viruses in untreated wastewater. Comparison of these concentrations from dry weather and under rainfall diluted ingress conditions provided an additional line of evidence on the dilution of influent between dry and wet weather to assess organic chemical results against. Additional MST samples were collected in 2022 and 2023 from both the sewer and receiving waters of the four locations as single or replicate samples (as listed in Table 4). After autosampler collection samples were transferred into 1250 mL PET bottles with unique laboratory traceability numbers, each being filled from two discrete refrigerated autosampler carousel bottles. Those sample containers were stored on ice in an Esky cooler at approximately 4 °C and delivered to the Sydney Water laboratory.

Additional samples of influent and receiving waters were collected for ammonia and metal analysis between February 2022 and March 2023, again using refrigerated autosamplers.

### Sample preparation and analysis

#### Organics

Prior to ultrahigh-performance liquid chromatographic analysis (UHPLC-MS/MS) at the Sydney Water laboratory, water samples were processed as outlined in Allinson et al. (2023) with 200 mL water samples filtered with glass-fibre filters (Whatman GF/C) before being loaded onto Oasis HLB cartridges (preconditioned with 5 mL methanol and 10 mL water; 200 mL at 10 mL min^−1^). The Oasis HLB cartridges used to extract the water samples were eluted with the methanol that was used to extract residues from the GF/C filter papers. The eluent was concentrated to around 400 μL by evaporation with nitrogen then internal standard mix was added. After reconstitution to 500 μL with methanol, samples were filtered with Millipore Millex-LG (conditioned with methanol). The National Association of Testing Authorities (Australia) has undertaken periodic audits of the Sydney Water facility since 1952.

All analyses involved appropriate QA/QC protocols including the use of internal standards of pure compounds carried through the full extraction and analysis procedures to determine recoveries. Respective suppliers and compound numbers of internal standards are detailed in Table 1. These calibration standards were diluted from stock solutions of each chemical following the procedures outlined in USEPA (2007). The standards were run after every 10 samples. No significant matrix effects were seen with recoveries of internal standards in the range of 80–114% (Table 1). Peaks were quantified from a 5-point calibration curve based on direct standard additions of the pure compounds in methanol to the instrument. Preparation blanks and sample duplicates were run with all analyses.

Two in-house UHPLC-MS/MS methods were used for the detection of organic contaminants. The first of these (method TC0063) enabled the detection of three benzotriazoles. This was based on the methods published by Li et al. (2017) and Zhang et al. (2011). The reconstituted extracts were diluted ten times prior to UHPLC-MS/MS analysis using the instrument conditions and reaction monitoring information described in Supporting information Tables S2 and S3, respectively.

The detection of a range of pharmaceuticals and personal-care products and other organic contaminants (as shown in Table 1) used in-house method TC0065. This was based on the ABSciex application note developed by Borton and Olson (2010), the Sciex application note by Lock (2011), ThermoFisher Scientific application note 20675 by MacRitchie and Philips (2013), the published method of Kaufmann et al. (2002), and USEPA (2007) method 1694. For this analysis, the reconstituted extracts were diluted ten times prior to UHPLC-MS/MS using instrument conditions and multiple reaction monitoring information described in Supporting information Tables S4 and S5, respectively.

For both methods, the analysis was performed using Sciex UHPLC-MS/MS in ESI mode comprising a Sciex Exion ultrahigh-performance liquid chromatograph interfaced with a Sciex QTRAP 6500 plus mass spectrometer. The analytical column used was Waters Acquity UHPLC BEH C18 column 2.1 mm × 100 mm, 1.7 μm (or equivalent). Analytical quantitation limits for organic chemicals in both methods are detailed in Table 1. Quantification limits were determined by the concentration equivalent of the smallest resolvable peak for each compound and multiplying this value by 5.

#### Metals

The metal analysis procedure was based on USEPA method 200.8. Approximately 10 mL of sample was heated with nitric and hydrochloric acid in a plastic tube in a hot block. For filterable metals, a portion of sample was filtered through a 0.45-μm filter membrane before preparation. After preparation, the samples were analysed by inductively coupled plasma–mass spectrometry (ICP-MS). The ICP-MS calibration standards were diluted from metrologically traceable stock solutions. Quality control samples were run with all analyses. Analytical quantitation limits for metals are detailed in Table 1. Metal analysis quantification limits were defined as being 10 times the standard deviation of low-level spike replicates. Seven-point calibration curves were used. Average matrix spike recoveries ranged from 86 to 101%.

#### Ammonia

The ammonia analysis procedure was an in-house method based on APHA method 4500-NH3 H. Samples were kept refrigerated after collection and analysed within 48 h. Samples were filtered through a 0.45-μm filter membrane before analysis then analysed by flow injection analysis (FIA) using the phenate colorimetric chemistry. The FIA calibration standards were diluted from traceable ammonia stock solutions. Quality control samples are run with all analyses. The analytical quantitation limit for ammonia is given in Table 1. Ammonia quantification limits were defined as being 10 times the standard deviation of low-level spike replicates. Seven-point calibration curves were used. Matrix spike recoveries averaged 99%.

#### Microbial source tracking

MST is founded on the premise of leveraging host-associated molecular marker genes found in bacteria, protozoa, and viruses in the faeces of a diverse range of animal species, including humans (Harwood et al. 2014). These marker genes are identified through the implementation of sequence analysis or other molecular methods such as subtractive hybridisation (Dick et al. 2005; Gomi et al. 2022). Many quantitative PCR (qPCR)-based assays have been developed to detect and quantify faecal pollution source-specific marker genes in environmental waters. The duplicate MST assay of Ahmed et al. (2019) was used to analyse the human-associated marker genes Bacteroides HF183 and CrAssphage CPQ_056 in the influent samples collected during diluted rainfall ingress conditions within the sewer carriers. Laboratory analysis of dry weather concentrations of these two MST markers is described by Ahmed et al. (2022).

## Results and discussion

### Detected chemicals

A total of only 18 compounds were found in concentrations above quantitation limits in autosampler-collected samples from UHPLC-MS/MS analysis, as listed in Table 2. This suite of compounds was analysed for in sewer influent, both in dry weather and in wet weather under rainfall ingress conditions when overflow spills were occurring from the ERSs (Table 2). Not surprisingly, the greatest number of detectable contaminants was seen in the dry-weather samples, where all 18 organic chemicals were in the autosampler-collected influent samples. The detected compounds had the following order of concentration: acetaminophen > metformin > theobromine > sucralose >ibuprofen > three benzotriazoles, with others close to quantitation limits. In rare instances, elevated concentrations of p-toluene sulfonamide were seen (Table 2). Under wet-weather conditions, the same order prevailed with only 12 of the dry-weather chemicals detectable (Table 2). A very similar pattern of chemical concentrations was seen in receiving waters downstream of ERSs when wet-weather overflow spills were occurring (Table 2). In the receiving waters, the number of detectable chemicals reduced to those 8 chemicals listed above in the highest concentrations.

**Table 2:** Summary of organic chemicals detected in sewer influent in dry weather and under wet-weather rainfall ingress dilution conditions, and in receiving waters downstream of ERS overflow spills with wet weather sample collection coinciding with overflow spills across the four sewer locations (Vineyard, Buffalo, Darling Mills, and Gymea) between October 2018 and February 2020

	Concentration, μg/L					*N*
Influent sampled under dry weather conditions	Minimum	Median	75th percentile	90th percentile	Maximum	
Acetaminophen	17	125	178	181	181	7
Atenolol	< 1	< 1	1	1	1	7
BP-3 (benzophenone-3)	< 1	< 1	1	1	1	7
Cotinine	< 1	1	2	2	2	7
Diclofenac	< 1	< 1	1	1	1	7
FB351	< 1	2	2	3	3	7
Hydrochlorothiazide	< 1	2	3	3	3	7
Ibuprofen	2	9	9	10	10	7
Metformin	18	56	72	72	72	7
Naproxen	< 1	2	3	3	3	7
Sotalol	< 1	2	2	2	2	7
Sucralose	8	23	25	26	26	7
Sulfapyridine	< 1	< 1	1	2	2	7
Theobromine	6	40	42	44	44	7
1H-benzotriazole	0.9	2.4	3	3.1	3.1	7
4-Methyl-1H-benzotriazole	0.9	2	2.3	2.3	2.3	7
5-Methyl-1H-benzotriazole	0.9	1.9	2.3	2.3	2.3	7
p-Toluenesulfonamide	< 1	< 1	1	91	91	7
Sewer influent sampled under diluted rainfall ingress conditions when overflow spills were occurring from ERSs	Minimum	Median	75th percentile	90th percentile	Maximum	*N*
Acetaminophen	< 1	21	38	54	148	123
Cotinine	< 1	< 1	< 1	< 1	2	123
FB351	< 1	< 1	1	1	2	50
Ibuprofen	< 1	1	2	4	13	123
Metformin	< 1	12	18	27	41	85
Naproxen	< 1	< 1	< 1	1	3	123
Sucralose	< 1	1	3	5	11	123
Sulfapyridine	< 1	< 1	< 1	< 1	2	123
Theobromine	< 1	6	9	15	26	123
1H-benzotriazole	< 0.25	0.25	0.7	1.1	3.6	123
4-Methyl-1H-benzotriazole	< 0.25	< 0.25	0.5	0.7	2.9	123
5-Methyl-1H-benzotriazole	< 0.25	< 0.25	0.5	0.7	2.5	123
Receiving water sampled downstream of ERSs during wet weather when overflow spills were occurring	Minimum	Median	75th percentile	90th percentile	Maximum	*N*
Acetaminophen	< 1	< 1	< 1	8	98	182
Ibuprofen	< 1	< 1	< 1	< 1	3	182
Metformin	< 1	< 1	< 1	< 1	11	142
Sucralose	< 1	< 1	< 1	1	7	182
Theobromine	< 1	< 1	< 1	3	10	182
1H-benzotriazole	< 0.25	< 0.25	< 0.25	0.4	3.0	182
4-Methyl-1H-benzotriazole	< 0.25	< 0.25	< 0.25	< 0.25	0.8	182
5-Methyl-1H-benzotriazole	< 0.25	< 0.25	< 0.25	< 0.25	0.8	182

^a^Detection limits of 0.25 μg/L for three benzotriazoles, and 1 μg/L for the other contaminants applied in calculation of these distributions; *FB351*, disodium distyrylbiphenyl disulfonate; *N*, number of samples

The presence of these is consistent with a known high usage of pharmaceuticals and personal-care products, in particular, acetaminophen (paracetamol) as a very common analgesic and metformin (antidiabetic) have been consistently listed in the top ten prescribed drugs in Australia (ASM 2020) without consideration of over-the-counter availability. Measured also were theobromine from chocolate and sucralose, used as a sweetener are prominent dietary constituents, while detected ibuprofen (Nurofen) is a common anti-inflammatory. The applications of benzotriazoles as corrosion inhibitors in dishwashing detergents (Shi et al. 2019), as ultraviolet filters in personal-care products (Zhao et al. 2017), along with their incorporation into textiles (Liu et al. 2017) resulted in their detection in sewage, while p-toluene sulfonamide a widely used plasticiser was also found.

In addition to organics, overflows will also contain metals, and importantly, ammonia. The challenge is to determine the likely downstream impact on ecosystem health that these contaminants may cause, taking into account the inevitable dilutions that they will be subjected to. For this exercise, it is important to have a marker chemical, present in the discharge that can be used to evaluate the extent of dilution. Then, with a knowledge of the potential toxicity of the discharged chemicals, it will be possible to determine whether the dilution is sufficient to reduce the concentrations of the most toxic of these to below those known to cause effects on aquatic biota.

### Comparison of detected contaminants across the four sewer carriers

The combined sewer site comparison in Table 2 summarises information presented in Table 3 for each sewer site. For each of the four sewer locations, the concentrations of individual contaminants and their percentile distributions of individual contaminant concentrations were relatively similar; however, as noted earlier, concentrations of individual contaminants differed substantially (Table 3). For example, acetaminophen was consistently present in the highest concentrations but concentrations at the Darling Mills sewer were considerably lower than at the other three (Table 3). On the other hand, metformin and theobromine concentrations were relatively similar at all four sites (Table 3). Sucralose and benzotriazoles at lower concentrations were also similar across sites (Table 3). Cotinine, FB351, naproxen, and sulfapyridine were all close to quantitation limits and trends were less obvious.

**Table 3:** Percentile distribution of contaminant concentrations (μg/L) of influent collected within sewer carrier when a spill was occurring from ERSs between October 2018 and February 2020

	Contaminant concentration, μg/L															
Contaminant	Acetaminophen				Cotinine				FB351				Ibuprofen			
	Vineyard	Buffalo	Darling Mills	Gymea	Vineyard	Buffalo	Darling Mills	Gymea	Vineyard	Buffalo	Darling Mills	Gymea	Vineyard	Buffalo	Darling Mills	Gymea
Minimum	< 1	2	< 1	<1	< 1	< 1	< 1	< 1	.	< 1	.	< 1	< 1	< 1	< 1	< 1
Median	27	27	< 1	21.5	< 1	< 1	< 1	< 1	.	< 1	.	< 1	1	< 1	< 1	2
75th percentile	44	38	7	44	< 1	< 1	< 1	< 1	.	< 1	.	1	2	< 1	< 1	4
90th percentile	49	88	11	60.5	< 1	< 1	< 1	< 1	.	1	.	1	3	1	1.5	6
Maximum	61	120	12	148	< 1	< 1	< 1	2	.	2	.	2	4	5	5	13
Contaminant	Metformin				Naproxen				Sucralose				Sulfapyridine			
	Vineyard	Buffalo	Darling Mills	Gymea	Vineyard	Buffalo	Darling Mills	Gymea	Vineyard	Buffalo	Darling Mills	Gymea	Vineyard	Buffalo	Darling Mills	Gymea
Minimum	8	< 1	3	4	< 1	< 1	< 1	< 1	< 1	< 1	< 1	< 1	< 1	< 1	< 1	< 1
Median	16	8	9	12.5	< 1	< 1	< 1	< 1	3	< 1	< 1	2	< 1	< 1	< 1	< 1
75th percentile	23	11	17	19.5	< 1	< 1	< 1	1	4	< 1	1.5	3	< 1	< 1	< 1	< 1
90th percentile	27	18	19.5	34.5	< 1	< 1	1.5	1	6	1	2	6	< 1	< 1	< 1	< 1
Maximum	30	27	22	41	1	2	3	2	8	4	7	11	< 1	< 1	< 1	2
Contaminant	Theobromine				1H-benzotriazole				4-Methyl-1H-benzotriazole				5-Methyl-1H-benzotriazole			
	Vineyard	Buffalo	Darling Mills	Gymea	Vineyard	Buffalo	Darling Mills	Gymea	Vineyard	Buffalo	Darling Mills	Gymea	Vineyard	Buffalo	Darling Mills	Gymea
Minimum	1	< 1	< 1	2	< 0.25	< 0.25	< 0.25	< 0.25	< 0.25	< 0.25	< 0.25	< 0.25	< 0.25	< 0.25	< 0.25	< 0.25
Median	5.5	5	2	10	0.4	0.3	< 0.25	0.4	< 0.25	< 0.25	< 0.25	0.4	< 0.25	< 0.25	< 0.25	0.4
75th percentile	7	5	5.5	14.5	0.8	0.6	0.4	0.7	< 0.25	0.4	0.6	0.6	< 0.25	0.4	0.6	0.6
90th percentile	9	6	8	18	1.8	1.2	0.5	1	0.5	0.5	0.8	0.8	0.5	0.5	0.8	0.8
Maximum	16	20	10	26	3.6	2.7	0.7	2	1.2	0.6	2.9	2	1.3	0.6	2.5	2

Detection limits values of 0.25 μg/L for three forms of benzotriazole and 1 μg/L for other nine contaminants applied in distribution calculation; Vineyard 38 samples were collected with standards not to hand for laboratory analysis of FB351 (disodium distyrylbiphenyl disulfonate) and for 28 samples of metformin; Buffalo 25 samples were collected and analysed for all 12 contaminants; Darling Mills 20 samples were collected with standards not to hand for laboratory analysis of FB351 and for 10 samples of metformin; Gymea 40 samples were collected and analysed for 11 contaminants while 15 samples were not analysed for FB351

As described above for Table 2, of the 12 contaminants detected in the rainfall ingress diluted influent, only eight were at measurable concentrations in the downstream receiving waters (as summarised in Table 2) when sampling coincided with spilling ERSs during wet weather. A view across the five receiving water sites revealed a contrasting pattern of organic chemical concentrations (Supporting information Table S6) compared to the relatively uniform pattern observed across the four sewer carriers when samples were collected under diluted rainfall ingress conditions (Table 3).

Analysis of receiving water samples from the Darling Mills site and from both Gymea Bay receiving water sites provided results at the quantitation level except for 1H-benzotriazole as shown for Gymea Bay Site 1 in Supporting information Table S6. Four contaminants (ibuprofen, metformin, sucralose, and theobromine) were only at measurable concentrations in samples from the Vineyard receiving water site (Supporting information Table S6). The other four chemicals, acetaminophen and the three benzotriazoles, were above quantitation limits in both Buffalo and Vineyard receiving water samples (Supporting information Table S6). Concentrations of the benzotriazoles were relatively similar between Buffalo and Vineyard Creeks, while acetaminophen was at relatively higher concentrations in Vineyard Creek (Supporting information Table S6).

The above data patterns may be partly explained by the placement of downstream receiving water sites. The Buffalo and Darling Mills sites had the same carrier diameter (Supporting information Table S1), while safe access considerations influenced the placement of the Buffalo site 50 m downstream of the ERS, compared to the more distant placement at Darling Mills some 950 m downstream (Fig. 1). At Gymea Bay, site placement catered for the two urban streams that drain into this estuarine bay, that both receive sewer overflows (Fig. 1). Hence, a site was placed at the mouth of each stream within the bay, with the nearest ERS to each bay arm situated at 400 m and 150 m to these stream mouths (Fig. 1). The ERS with the relatively highest modelled volume for the Gymea study location (Supporting information Table S1) was nearest to Gymea Site 2. All ERSs under study at the Buffalo, Darling Mills, and Gymea locations were gravity fed. Safe access to Vineyard Creek occurred 140 m downstream from three ERSs that discharged within 10 m into the same waterway reach (Fig. 1, Supporting information Fig. S1). The highest modelled volume ERS at this location was a siphonic overflow discharge structure (Supporting information Table S1) that conveyed spills from the 2100-mm diameter sewer main, while on the opposite side of the creek the other two ERSs were gravity fed and spilt from the 1830-mm sewer main, although a small connecting pipe allowed one of these two ERSs to also spill influent from the 2100-mm sewer main. Hence, the Vineyard location was atypical with three ERSs situated very close together (as evident in Supporting Fig. S1) compared to the other three study locations where those sewer trunk and associated reticulation pipes that drained urban areas had ERSs situated at disparate distances along the sewerage system (Fig. 1).

In addition to the distance of the receiving water site from the ERS source of contaminants contained in overflow spills, the associated spill volumes from an overflow should be considered in the contribution to dilution achievable in the receiving waters along with rainfall from respective wet-weather events. Wet-weather overflow median spill volumes from October 2018 to October 2022 were 0.04 ML (range 0.0001 to 11.3 ML over 36 spills) from the Gymea sewer, 0.3 ML (range 0.004 to 41 ML over 48 spills) from the Buffalo sewer, 4.8 ML (range 0.03 to 146 ML over 31 spills) from the Darling Mills sewer, and 7.6 ML (range 0.001 to 1159 ML over 55 spills) from the Vineyard sewer.

Supporting information Table S7 shows the percentage of collection events (occasions) where the eight organic chemicals were detected in waters from each of the five receiving water sites. No measurable concentrations were recorded for any of the eight organic chemicals at Gymea Site 2 or at the Darling Mills receiving water sites, while only 1H-benzotriazole was measurable in about half of the collection events at Gymea Site 1 receiving water site. At the Buffalo receiving water site, acetaminophen was measurable in two of the five collection events as was 1H-benzotriazole, while 4-methyl-1H-benzotriazole and 5-methyl-1H-benzotriazole were measured in one of the collection events. In contrast, all eight organic chemicals were detected at the Vineyard receiving water site, although the number of events in which measurable concentrations were detected varied from once for 4-methyl-1H-benzotriazole, 5-methyl-1H-benzotriazole, and ibuprofen to all eight sample occasions for theobromine (Supporting information Table S7).

Overflow spill volumes do not appear to explain the detection of measurable concentrations in the receiving waters. For example, a relatively higher median spill volume occurred into Darlings Mills Creek (4.8 ML) when compared against the relatively lower median spill volume into Buffalo Creek (0.3 ML). The opposite pattern to that recorded for detection of measurable organic chemical concentrations would have been expected for these two sites. A more plausible explanation is the dilution over distance (50 m for Buffalo versus 950 m for Darling Mills) from an ERS source of a wet weather overflow spill, as the explored patterns of measurable concentration detection across sample events seem supportive given there were no measurable detections for any of the eight organic chemicals at Darling Mills, while four organic chemicals were recorded at measurable concentrations in the receiving waters of the Buffalo site (Supporting information Table S6).

### Identifying suitable sewage marker chemicals

Of the organic chemicals assessed, none were detected in receiving water samples collected under light rainfall conditions where autosamplers were triggered after 2 mm of rainfall occurred in 15 min. This lack of detection, together with gauging of ERSs that recorded no sanitary sewer overflows to have occurred during these light rainfall events, suggests that the origin of these contaminants is from disposal into the sewer. As such, these organic contaminants provide a line of evidence to track wet-weather sanitary sewer overflows. The eight chemicals detected in the receiving waters during overflow spill events (Table 2 and Supporting information Table S6) as well as in both dry-weather influent and in wet-weather ingress diluted influent are potentially more sensitive marker chemicals to indicate sewage contamination (Table 2).

Three of the eight potentially more sensitive organic chemicals of the current study were also employed as tracers of sewage in other studies. For example, Currens et al. (2019) used acetaminophen and sucralose as co-analytes to track sewage in receiving waters, while White et al. (2019) employed acetaminophen and ibuprofen to trace untreated sewage inputs in receiving waters and determined sucralose to be an excellent tracer of both treated and untreated sewages. The suitability of sewage-marker chemicals may, however, be limited by the short half-lives of some chemicals in the receiving waters.

Acetaminophen is an immediate chemical of interest, with concentrations in the overflows as high a 148 μg/L. The half-life for acetaminophen in river waters due to photolysis has been reported as between 56 and 77 h (Yamamoto et al. 2009) with a similar biodegradation half-life (50 h), indicating that it should persist in a river system between discharge and dilution. The rate of formation of any more toxic degradation products would therefore be slow.

Few data are available for the half-lives of other overflow constituents in fresh waters. Of those found from a literature search, the in-stream decay half-life for metformin was 1 day (Caldwell et al. 2019) and this is a high usage pharmaceutical, which suggests this may be a suitable marker of sewage (Balakrishnan et al. 2022).

Shi et al. (2019) described benzotriazoles as usually showing high detection rates (> 95%) both in wastewater influent and effluent samples. As outlined above, sources of benzotriazoles within influent are from the applications of benzotriazoles as corrosion inhibitors in dishwashing detergents, ultraviolet filters in personal-care products, along with their incorporation into textiles. In the current study, three benzotriazoles were included in the eight organic chemicals measured in receiving water and influent samples (Table 2). Benzotriazoles are also used in cooling and brake fluids (Shi et al. 2019), and as vulcanisation accelerators in rubber production with tyre-wear particles identified in both road dust and roadside soil (Zhang et al. 2018), which may all be sources within stormwater runoff. However, in the current study, detectable concentrations were not recorded in samples collected during storm events that did not coincide with sewer overflow spills. This suggests that the three benzotriazoles tracked in the current study are suitable marker chemicals to track sewage contamination in receiving waters. Chung et al. (2018) reported photolysis half-lives of 44 and 25 h, respectively, for 1-H-benzotriazole and 5-methyl-1H-benzotriazole.

Theobromine is a component of chocolate, tea, and cocoa as well as being used in formulations of pharmaceuticals and personal-care products. It is also a caffeine metabolite (Martínez-Pinilla et al. 2015). It appears to be a suitable marker due to its widespread consumption although the half-life is not known.

Consideration should also be given to processes that might modify the potential bioavailability from the form in which it is discharged. Most toxic effects are due to dissolved contaminants; however, processes that involve the partitioning of dissolved forms to suspended particulates and degradation processes such as hydrolysis or photolysis will together reduce their bioavailability over and above that predicted by dilution. Downstream measurements could therefore result in an overestimation of the dilution under such cases in unfiltered water samples. A complicating factor can be if the degradation products are more toxic than the parent compounds. The measured target compounds of the current study as well as unmeasured compounds in the water column may be subject to the above processes as influences on toxicity.

### Evaluating dilutions

A comparison of median concentrations of organic contaminants in sewer influent collected in dry weather and under wet-weather rainfall ingress diluting conditions that coincided with overflow spills had a relatively tight range of initial dilutions from 4.9× to 9× for five of six contaminants assessed (Table 4). The sixth contaminant, sucralose, exhibited a dilution of 23× (Table 4). The companion line of evidence that compared dry- to wet-weather concentrations of human-associated MST marker genes measured in sewer influent returned dilutions of 3.8× for Bacteroides HF183 and 12.4× for CrAssphage CPQ_056 (Table 4), which were supportive of dilutions determined from organic contaminants.

**Table 4:** Dilutions of organic chemicals and human-associated MST marker genes measured in sewer influent collected from ERSs in dry weather and under wet weather rainfall ingress diluted conditions that coincided with overflow spills for samples collected from all four sewer sites across all collection events between October 2018 and February 2020

	Dry weather median	Wet weather median	Dilution^a^
Organic chemicals	μg/L	μg/L	Dry/wet
Acetaminophen	125	21	6.0×
Ibuprofen	9	1	9.0×
Metformin	56	11.5	4.9×
Sucralose	23	1	23.0×
Theobromine	40	6	6.7×
1H-benzotriazole	2.4	0.3	8.0×
Number of samples	7	123	
Human associated MST marker	Dry weather	Wet weather	Dilution^b^
	geometric mean (log10 GC/L)	geometric mean (log10 GC/L)	
CrAssphage CPQ_056	9.18	8.08	12.4×
Bacteroides HF183	9.03	8.45	3.8×
Number of samples	72	18	

^a^Dilutions based on medians

^b^Dilutions based on back-transformed geometric means

*GC/L*, gene copies/L

For wet weather overflows, companion measurements of human-associated MST marker genes collected during wet-weather overflow spills on February 22 and March 1, 2022, from Darling Mills and Buffalo locations provided evidence of dilution in the receiving waters. A single sample was collected from the wet-weather diluted ingress influent and from the receiving water at each location. The Buffalo receiving waters were sampled 50 m downstream of the spilling ERS returned dilutions of 4.9 times for Bacteroides HF183 and 6.4 times for CrAssphage CPQ_056 (Table 5). Higher dilutions were observed at the more distant receiving water location for Darling Mills, some 950 m downstream of the ERS, with dilutions of 28 times for Bacteroides HF183 and 65 times for CrAssphage CPQ_056 (Table 5).

**Table 5:** Dilutions of organic chemicals and human-associated MST marker genes from measurements in downstream receiving waters (RW) and sewer influent collected under wet weather rainfall ingress diluted conditions that coincided with overflow spills from Darling Mills, Buffalo, and Vineyard sewer carriers via ERS

Contaminant	Darling Mills			Darling Mills			Buffalo			Buffalo		
	February 2, 2022			January 24, 2023			March 1, 2022			July 4, 2022		
	Influent	RW	Dilution	Influent	RW	Dilution	Influent	RW	Dilution^a^	Influent	RW	Dilution^a^
Ammonia, mg total ammonia N/L	1.9	0.1	19×	2.79	0.1	28×	0.8	0.2	4×	0.8	0.2	4×
Acetaminophen, μg/L	< 1	< 1	-	ns	ns	-	6	< 1	> 6×	ns	ns	-
Metformin, μg/L	12	< 1	> 12×	ns	ns	-	13	2	6.5×	ns	ns	-
Theobromine, μg/L	3	< 1	> 3×	ns	ns	-	2	< 1	> 2×	ns	ns	-
Number of samples	1	1		5	5		1	1		7	7	
Human-associated MST marker	Geometric mean			Geometric mean			Geometric mean					
	Influent	RW	Dilution^b^	Influent	RW	Dilution^b^	Influent	RW	Dilution^b^	Influent	RW	Dilution^b^
	log10 GC/L		log10 GC/L		log10 GC/L							
CrAssphage CPQ_056	8.70	6.89	65×	8.38	6.47	81×	8.63	7.83	6.4×	ns	ns	-
Bacteroides HF183	8.49	7.04	28×	8.32	6.57	57×	8.77	8.08	4.9×	ns	ns	-
Number of samples	1	1		2	2		1	1				
Contaminant	Vineyard			Vineyard			Vineyard					
	October 5, 2022			October 6, 2022			October 9, 2022					
	Influent	RW	Dilution^a^	Influent	RW	Dilution^a^	Influent	RW	Dilution			
Ammonia, mg total ammonia N/L	9.4	2.3	4.1×	4.9	5.6	0.9×	1.1	1.0	1.1×			
Acetaminophen, μg/L	ns	ns	-	ns	ns	-	< 1	< 1	-			
Metformin, μg/L	ns	ns	-	ns	ns	-	38	33	1.2×			
Theobromine, μg/L	ns	ns	-	ns	ns	-	1	< 1	> 1×			
Number of samples	5	5		5	5		1	1				
Human-associated MST marker	Geometric mean			Geometric mean			Geometric mean					
	Influent	RW	Dilution^b^	Influent	RW	Dilution^b^	Influent	RW	Dilution^b^			
	log10 GC/L			log10 GC/L			log10 GC/L					
CrAssphage CPQ_056	8.91	8.61	2.0×	8.73	8.82	0.8×	7.99	7.91	1.2×			
Bacteroides HF183	9.02	8.74	1.9×	8.98	9.02	0.9×	8.36	8.20	1.4×			
Number of samples	2	2		2	2		1	1				

^a^Dilutions based on mean values

^b^Dilutions based on back-transformed geometric means

*GC/L*, gene copies/L, *ns*, not sampled

During the weather event of July 4, 2022, a mean concentration of 0.2 mg total ammonia N/L (*n* = 7) was recorded in the receiving water downstream of the Buffalo ERS (Table 5), in samples collected while an overflow spill occurred. Companion measurement in the influent during this event recorded 0.8 mg total ammonia N/L (*n* = 7) (Tables 5 and 6). These influent and receiving water results mirrored total ammonia N/L from March 1, 2022 (Table 5), and suggested that over the 50 m distance from the ERS to the downstream autosampler, a dilution of 4× had occurred in the receiving waters at the Buffalo location (Table 5). This dilution of 4× is supportive of the above-mentioned MST results (Table 5) as was the metformin dilution of 6.5 times recorded at this location (Table 5). The trend of relatively higher dilutions observed in MST results recorded at the more distant receiving water location of Darling Mills (Table 5) was also observed for total ammonia N/L where dilutions of 19 and 28 times were recorded for the February 2, 2022, and January 24, 2023, samples, respectively (Table 5). These diluted ammonia concentrations measured in the receiving waters for both the Buffalo and Darling Mills sites were at least four times less than the ANZG (2018) water quality guideline value (0.75 mg total ammonia N/L). This suggests that the risk to ecosystem health posed by influent concentrations of ammonia was abated by dilution of influent in receiving waters at the distances that sites were situated in the current study.

**Table 6:** Concentrations of metals and ammonia measured in water column influent of sewer carriers at Vineyard, Darling Mills, and Buffalo compared to ANZG (2018) water quality guideline values

Contaminant	Vineyard dry weather^a^ (January 21, 2021)	Vineyard wet weather ^b^ (May 6, 2021)	Vineyard wet weather ^b^ (July 4, 2022)	Vineyard wet weather ^b^ (October 5, 2022)	Vineyard wet weather ^b^ (October 6, 2022)	Vineyard wet weather ^b^ (October 9, 2022)	Darling Mills wet weather ^b^ (February 22, 2022)	Buffalo wet weather ^b^ (March 1, 2022)	Buffalo wet weather ^b^ (July 4, 2022)	Guideline value (95% species protection)^d^
Ammonia, mg total ammonia N/L	62.6	15.0	4.2	9.4	4.9	1.1	1.9	0.8	0.8	0.75 mg total ammonia/L
Aluminium dissolved, μg/L	0.8	28	112	129	91	51	73	92	95	55
Iron total, μg/L	6720	3930	1340	2050	1840	1650	1410	1250	1800	700
Iron dissolved, μg/L	577	214	358	431	393	234	212	204	258	-
Manganese dissolved μg/L	106	36	55	58	52	29	37	9	11	1900
Copper dissolved, μg/L	5.9	4.9	10.1	8.3	8.5	6.2	7.2	11.4	11.1	1.4
Zinc dissolved, μg/L	10	14	22	26	24	27	26	26	24	8
Lead dissolved, μg/L	0.1	0.2	0.4	1.0	2.2	0.3	0.3	0.8	0.9	3.4
Cadmium dissolved, μg/L	< 0.1	< 0.1	< 0.1	< 0.1	< 0.1	< 0.1	< 0.1	< 0.1	< 0.1	0.2
Selenium total, μg/L	0.7	0.5	0.3	0.3	0.3	0.3	0.4	0.4	0.3	11
Rainfall, mm in 120(24) hours	0(0)	57 (19)	154 (31)	31 (22)	43 (20)	96 (46)	116 (89)	126 (35)	164 (32)	-
Spill volume, ML	0^a^	0^c^	188.2	3.8	29.2^e^	110.2^f^	10.4	1.2	16.0	-

^a^Dry weather conditions without rainwater ingress into the sewerage system

^b^Under wet weather conditions with rainwater ingress into the sewerage system

^c^Sampling was inadvertently triggered off spill from ERS on adjacent sewer main that intersects downstream of sewer main that sewer autosampler was situated. No discharge was recorded from sewer main with autosampler, while 1.9 ML was recorded from the adjacent sewer main

^d^From ANZG (2018)

^e^Cumulative volume of October 5 and 6, 2022

^f^Cumulative volume of October 5, 6, and 9, 2022

The ERSs under study at the Buffalo and Darling Mills locations were gravity fed with influent transported by their respective sewer main pipes from the local urban area, with the Buffalo and Darling Mills locations being more representative of urban areas in Sydney where spatially separated ERSs spill to freshwater urban streams (Fig. 1). In contrast, the Vineyard location is atypical of the local sewerage system with the merger of trunk main pipes that transport influent from a larger geographic area and three ERSs that spill within 10 m to the same reach of a small urban stream (Fig. 1, Supporting information Fig. S1), with one of these ERSs capable of siphonic discharge flows. The siphonic discharge is initiated by a gravity flow, which then primes a siphonic pipe that accelerates the discharge spill rate. Examples of siphonic and gravity-fed spill rates are available in Besley and Cassidy (2021). A siphonic discharge continues until the flow falls low enough within the sewer pipe to break the siphonic flow. As such, a siphonic overflow spill can continue to occur after rainfall has ceased in the area. The combination of the accelerated siphonic flows from one ERS together with the two-gravity fed ERS spills at the same point into this relatively small urban stream may help explain the limited receiving water dilutions of around 1 observed at this location on October 6 and 9, 2022 (Table 5). In contrast, at the start of the October weather event on October 5, 2022, dilutions of two to four times were recorded for MST marker genes and ammonia (Table 5). This suggests that later in a protracted spill event at the Vineyard location, the flow in the receiving waters of Vineyard Creek comprised mostly of rainwater ingress diluted influent. Sampling of these three collection events across this wet-weather period illustrated that the influent within the sewer pipe became most highly diluted from rainwater ingress as cumulative rainfall increased (Table 6). A discussion of cumulative rainfall for this October 2022 weather event is provided below. This in turn suggests that the ecological risk posed in receiving waters from ammonia diminished as spill duration from the ERSs increased. An ecological risk was potentially present from recorded ammonia concentrations in receiving waters due to the relatively low dilution confirmed by estimates from companion lines of evidence (Table 5). The few other organic chemicals with detected concentrations in the sewer influent were not present in measurable concentrations within the receiving waters, and as such did not yield reliable dilution values (Table 5).

The receiving waters were also sampled from two sites of Gymea Bay and from the associated sewer main on October 6, 2022, after the target ERS commenced spilling along with two other, spatially separated ERSs that also spill into the same creek which flows into the bay at Gymea Bay Site 2. These ERSs commenced spilling within 15 min of each other and continued for 1 h to 2.5 h. Very high receiving water dilutions of 130 to 172 times were suggested from results for ammonia and from MST marker genes with over 10,000 times dilution. Another wet-weather overflow event was sampled on January 6, 2023, after a single ERS spilt for 4.25 h into the creek that flows into the bay at Gymea Bay Site 2. Very high receiving water dilutions were again observed, with over 300 times for ammonia and from over 500 to 15,000 times for MST marker genes. A third wet-weather overflow event was captured on February 6, 2023, when the seven ERSs (Fig. 1) in this sewer catchment spilled for 2.7 h in response to an intense storm event that deposited 30 mm of rainfall in 1 h. The relatively highest receiving water concentration of ammonia at 0.07 mg (total ammonia N/L) was recorded in samples collected from Gymea during this storm event. These samples represented the lowest receiving water dilution of 68 times at the Gymea study location. These outcomes support organic chemical results from Gymea Bay Site 1 and Gymea Bay Site 2 detailed in Supporting information Table S6, where 11 of 12 organic chemicals were not detected in the receiving waters, while 1H-benzotriazole was detected in two of 68 samples. This suggests that an ecological risk is potentially not posed by gravity-fed ERS spills into the tidally flushed Gymea Bay estuarine receiving water environment from the lines of evidence assessed in the current study.

### Toxicity of overflow chemicals and the risk of adverse ecological impacts

Assuming an absence of significant degradation pathways, it was important to review the available ecotoxicity data for the key contaminants in freshwaters. Again, there were limited data that have been summarised in Table 7. This table includes data for some compounds that were barely detected but were added for completeness. The most common organisms tested were the water flea *Daphnia magna*, and the microalga, *Raphidocelis subcapitata*, enabling comparisons between the different contaminants.

**Table 7:** Toxicity data for key sanitary sewer overflow contaminants that were measurable within influent

Contaminant	Use	Organism	Endpoint	Toxicity value, mg/L	Reference
Contaminants observed in influent within the sewer carrier water column under both dry and rainfall ingress diluted wet weather conditions					
Acetaminophen	Analgesic (paracetamol)		Acute HC5	7.6	Nunes et al. (2014)
		*Raphidocelis subcapitata*	Chronic 96-h EC10	91	Wang et al. (2015)
		*Daphnia magna*	Acute 48-h EC50	4.7	Nunes et al. (2014)
		*Daphnia magna*	Acute 48-h EC50	2.8	De Oliveira et al. (2016)
		*Daphnia magna*	Chronic 21-d NOEC	> 1.25	De Oliveira et al. (2016)
Cotinine	Nicotine metabolite	*Daphnia magna*	Acute 48-h EC50	0.169	Vlascianu et al. (2015)
Disodium distyrylbiphenyl disulfonate (FB351)	Detergent, optical brightener	*Daphnia magna*	Acute 48-h EC50	1000	MSDS (2015)
Ibuprofen	Anti-inflammatory	*Raphidocelis subcapitata*	Chronic 72-h NOEC	0.010	Brun et al. (2006)
		*Desmodesmus subspicatus*	72-h IC20	103 (342)	Cleuvers (2004)
		*Daphnia magna*	48-h EC50 (EC10)	66 (101)	Cleuvers (2004)
Metformin	Anti-diabetic	*Desmodesmus subspicatus*	Chronic 72-h IC50	> 320	Cleuvers (2003)
		*Daphnia magna*	48-h EC50	64	Cleuvers (2003)
Naproxen	Anti-inflammatory	*Raphidocelis subcapitata*	Chronic 96-h EC50	32	Isidori et al. (2005)
		*Ceriodaphnia dubia*	48-h EC50	6.6	Isidori et al. (2005)
		*Ceriodaphnia dubia*	Chronic 7-d EC50	0.33	Isidori et al. (2005)
		*Daphnia magna*	48-h EC50	30	Kwak et al. (2018)
		*Daphnia magna*	Chronic 21-d NOEC	10	Kwak et al. (2018)
		*Moina macrocopa (*crustacean)	Chronic 7-d NOEC	0.3	Kwak et al. (2018)
Sucralose	Artificial sweetener	*Raphidocelis subcapitata*	Chronic EC50	> 1800	Smyth (1986) in Tollefsen et al. (2012)
		*Daphnia magna*	48-h EC50	> 1800	Jenkins (1984) in Tollefsen et al. (2012)
		*Daphnia magna*	Chronic 7-d EC50	> 1800	Williams (1986) in Tollefsen et al. (2012)
Sulfapyridine	Antibacterial	*Raphidocelis subcapitata*	Chronic 72-h IC50	10.2	Blaise et al. (2006)
		*Hydra attenuata*	Chronic NOEC	1	Quinn et al. (2008, 2009)
Theobromine	Alkaloid in chocolate; caffeine degradation product	No data			
1H-Benzotriazole	Corrosion inhibitor	*Desmodesmus subspicatus*	Chronic EC10	1.2	Seeland et al. (2012)
		*Daphnia magna*	48-h EC50	107	Seeland et al. (2012)
4-Methyl-1H-benzotriazole	Corrosion inhibitor	No data			
5-Methyl-1H-	Corrosion inhibitor	*Desmodesmus subspicatus*	Chronic EC10	2.8	Seeland et al. (2012)
benzotriazole		*Daphnia magna*	48-h EC50	52	Seeland et al. (2012)
		*Daphnia magna*	Chronic 21-d EC50	5.9	Seeland et al. (2012)
Additional contaminants observed in dry weather influent					
Atenolol	β-blocker	*Desmodesmus subspicatus*	Chronic 72-h EC50	620	Cleuvers (2005)
		*Daphnia magna*	48-h EC50	313	Cleuvers (2005)
Benzophenone-3 (BP3)	Sunscreen agent	*Desmodesmus subspicatus*	Chronic 72-h EC10 (IC50)	0.61 (0.96)	Sieratowicz et al. (2011)
		*Daphnia magna*	48-h EC50 (EC10)	1.67 (1.24)	Sieratowicz et al. (2011)
		*Daphnia magna*	Chronic 21-d NOEC	0.5	Sieratowicz et al. (2011)
Diclofenac	Anti-inflammatory (Voltarin)		Chronic HC5	0.77	Kumar et al. (2016)
		*Daphnia magna*	48-h EC50	68	Cleuvers (2004)
Hydrochlorothiazide	Diuretic	*Raphidocelis subcapitata*	Chronic 72-h NOEC	100	Astra-Zenica (2017)
		*Daphnia magna*	Chronic 21-d NOEC	100	Astra-Zenica (2017)
p-Toluenesulfonamide	Plasticiser	No data			
Sotalol	β-blocker	*Daphnia magna*	48-h EC50	204	Khalit and Tay (2017)
		*Daphnia magna*	Chronic EC50	2530	Khalit and Tay (2017)

Ideally, the requirement is for chronic EC10 data for at least 8 species to enable derivation using a species sensitivity distribution (SSD), of the concentration in freshwaters hazardous to 5% of species (HC5) (ANZG 2018). This was available for one contaminant only, ibuprofen. For acetaminophen, an acute HC5 was reported (Table 7) (Nunes et al. 2014); however, this mistakenly combined acute data with chronic 72-h algal growth data (Warne et al. 2018).

It is noteworthy that several papers have reported predicted no effect concentration (PNEC) values for some of the contaminants. PNEC values are based on the application of large assessment factors to the most sensitive toxicity data and are inherently unreliable compared to SSD-derived guideline values (GVs). For example, Comber et al. (2018) reported PNEC values of 148 μg/L for atenolol, 0.05 μg/L for diclofenac, 0.01 μg/L for ibuprofen, and 13.5 μg/L for metformin. For comparison, Caldwell et al. (2019) reported a 75-times greater PNEC of 1 mg/L for metformin, derived by applying a factor of 10 to the lowest of chronic data for algae daphnids and fish. Note also, the reliable SSD-derived HC5 value of 77 μg/L for diclofenac (Table 7), which is some 3000 times the PNEC value.

Many of the toxicity data in Table 7 are in the mg/L range, and from an assessment of those data presented, only cotinine, ibuprofen, and naproxen might pose concerns if concentrations were to exceed around 10 μg/L. For ibuprofen, the 4 orders of magnitude greater sensitivity of the microalga *Raphidocelis subcapitata* compared to a similar green algae *Desmodesmus subspicatus* (Table 7) is difficult to accept and bears repeating.

Looking at the measured concentrations of the identified contaminants of potential concern in Table 2, it would appear that none of the chemicals are likely to pose concerns for ecosystem health before dilution, and even less likely after dilution in the receiving waters (Table 2) when compared to toxicity data outlined above. It should be noted that there were chemicals for which no toxicity data could be obtained, but these chemicals were not present in high concentrations in the overflow waters.

Concentrations of metals and ammonia measured in sanitary sewer overflows are shown in Table 6 and are compared to toxicity data. With the exception of copper and zinc, dissolved metal concentrations were below water quality guideline values (Table 6). The bioavailable concentration of copper and zinc will be significantly reduced by the high concentration of dissolved organic carbon in wet-weather samples of 73 mg/L (May 6, 2021) from Vineyard, 7.7 mg/L (February 22, 2022) from Darling Mills, 8.7 mg/L (March 1, 2022) from Buffalo, and 8.4 mg/L (October 9, 2022) from Vineyard under the much more rainfall ingress diluted influent conditions. The guideline value for iron, recently revised, is based on total iron because toxicity is due to both colloidal and particulate iron as well as dissolved iron as iron solubility is easily exceeded (ANZG 2018). Although iron concentrations in these samples exceeded the guideline value, there is evidence that it is likely to be complexed by natural organic ligands and not necessarily bioavailable or toxic (Nagai et al. 2007).

Of greater concern are the high ammonia concentrations (Table 6). The recently revised ammonia guideline value at pH 7 and 20 °C is 0.75 mg total ammonia N/L. The pH values of the dry and five wet weather samples were, respectively, 7.4, 7.2, 7.2, 6.9, 7.0, and 7.1 from the Vineyard sewer. The guideline value exceedances were, respectively, 84, 20, 6, 13, 7, and 1.5 times for the dry- and wet-weather samples. The May 6, 2021, wet-weather event (19 mm in 24 h to 9 am on day of sampling) sampled from Vineyard with 20 times exceedance of the guideline value is potentially representative of the least dilution under rainfall ingress conditions as sampling was conducted the day before an overflow spill occurred from this sewer main. Sampling was inadvertently triggered at an ERS situated on another sewer main that joins with this main. Samples in the second wet-weather event of July 4, 2022, from Vineyard were correctly collected after the nearest ERS was confirmed to be spilling. The lesser guideline value exceedance of 5.6 times reflects the potentially greater rainfall ingress that had occurred during this event as 154 mm of rain fell in 120 h to 9 am on day of sampling (Table 6), although the highest rainfall recorded between 2018 and July 2022 was 346 mm in February 2020 (over 120 h). Diminishing trends in ammonia and for aluminium, iron, and manganese were recorded in influent across the three wet-weather collection events in early October, 2022, from Vineyard (Table 6). The sample event conducted on October 5 was collected during the first hour of overflow spills that continued for 20 h into October 6, when the second collection was undertaken during the eighteenth hour of this continuous spill. The third collection on October 9 was also collected in the fourteenth hour of a 24-h spill that commenced in the evening of October 8, 2022. Cumulative rainfall totals increased across these three sample events with 20 mm, 43 mm, and 96 mm up to the respective sample times for these events from the start of this wet-weather period in the early hours of October 5, 2022. Increased rainwater ingress into the sewerage system is likely to have occurred during this period and may explain the diminishing trend recorded from these three sampling events conducted at the Vineyard location in October, 2022 (Table 6).

The lowest ammonia concentrations of 0.8 mg total ammonia N/L (pH of 7.0 and 6.9) measured in the Buffalo sewer influent under wet-weather ingress conditions on two occasions were just above the guideline value (of 0.75 mg total ammonia N/L) (Table 6). The low ammonia concentrations recorded in the Buffalo sewer were also approached in the highly diluted rainwater ingress influent of the Vineyard sewer on October 9, 2022, when an ammonia concentration of 1.1 mg total ammonia N/L was recorded (Table 6).

## Conclusions

Of the organic contaminants tracked, 18 were measurable in dry-weather influent, while 12 of those were relatively uniformly detected in influent under rainfall ingress diluted conditions across all four sewer study sites.

The lack of uniformity of detection across receiving water sites for the subset of eight organic chemicals detected during overflow spill events suggests that the use of organic chemicals in tracking sewage in receiving waters would be best performed using this suite of eight organic chemicals that comprised acetaminophen, ibuprofen, metformin, sucralose, theobromine, and three benzotriazoles.

The additional line of evidence to assess dilution provided by companion human-associated MST marker gene data was supportive of dilutions determined with the two lines of evidence from organic chemical and ammonia data.

While none of the organic chemicals tracked in wet-weather ingress-diluted influent posed concern for ecosystem health, the companion measurements of bioavailable concentrations of zinc and copper in wet-weather ingress diluted influent were above water quality guideline values. However, any concern for ecosystem health from these two metals was potentially ameliorated by relatively high dissolved organic carbon concentrations present in influent. In contrast, a risk to ecosystem health was posed by ammonia concentrations in influent, but this risk was abated by dilution in receiving waters at the distances at which sites were situated in the current study. However, in the relatively small urban stream of Vineyard Creek, the atypical sewer main carrier convergence where three ERSs, including a siphonic discharge, can spill influent to comprise most of the flow, particularly under longer spill durations, ammonia may pose a risk to ecosystem health due to the limited receiving water dilutions achieved. Ammonia concentrations were observed to diminish as cumulative rainfall totals increased.

## Supplementary Information

ESM 1 (DOCX 2282 kb)

## Data Availability

The authors do not have permission to share data. A reasonable request enquiry can be made to Sydney Water to seek a data sharing licence for data in this study.

## References

[CR1] Ahmed W, Payyappat S, Cassidy M, Besley C (2019). A duplex PCR assay for the simultaneous quantification of Bacteroides HF183 and crAssphage CPQ_056 marker genes in untreated sewage and stormwater. Environ Int.

[CR2] Ahmed W, Bivins A, Payyappat S, Cassidy M, Besley C (2022). Distribution of human fecal marker genes and their association with pathogenic viruses in untreated wastewater determined using quantitative PCR. Water Res.

[CR3] Allinson M, Cassidy M, Kadokami K, Besley C (2023). In situ calibration of passive sampling methods for urban micropollutants using targeted multiresidue GC and LC screening systems. Chemosphere.

[CR4] ANZG (2018) Australian and New Zealand guidelines for fresh and marine water quality. Australian and New Zealand Governments and Australian state and territory governments, Canberra ACT, Australia. Available at: www.waterquality.gov.au/anz-guidelines. Accessed 1 Dec 2022

[CR5] ASM (2020) Australian Statistics on Medicines. Available at https://www.pbs.gov.au/info/statistics/asm/australian-statistics-on-medicines

[CR6] Astra-Zenica (2017) Environmental risk assessment data hydrochlorothiazide. Available at https://www.astrazeneca.com/content/dam/az/our-company/Sustainability/2017/hydrochlorothiazide.pdf

[CR7] Balakrishnan A, Sillanpää M, Jacob MM, Vo D-VN (2022). Metformin as an emerging concern in wastewater: occurrence, analysis and treatment methods. Environ Res.

[CR8] Besley CH, Cassidy M (2021). The composition of gross pollutants contained in wet weather overflows for different locations, spill frequencies and discharge volumes. J Environ Manage.

[CR9] Blaise C, Gagne F, Eullaffroy P, Ferard J-F (2006). Ecotoxicity of selected pharmaceuticals of urban origin discharged to the Saint-Lawrence River (Quebec, Canada): a review. Braz J Aquat Sci Technol.

[CR10] Borton C, Olson L (2010) Analysis of endocrine disruptors, pharmaceuticals, and personal care products in river water. ABSciex Application Notes, Food & Environment

[CR11] Brun GL, Bernier M, Losier R, Doe K (2006). Pharmaceutically active compounds in Atlantic Canadian sewage treatment plant effluents and receiving waters, and potential for environmental effects as measured by acute and chronic aquatic toxicity. Environ Toxicol Chem.

[CR12] Caldwell DJ, D’Aco V, Davidson T, Kappler K, Murray-Smith RJ, Owen SF, Robinson PF, Simon-Hettich B, Straub JO, Tell J (2019). Environmental risk assessment of metformin and its transformation product guanylurea: II. Occurrence in surface waters of Europe and the United States and derivation of predicted no-effect concentrations. Chemosphere.

[CR13] Camargo JA, Alonso A (2006). Ecological and toxicological effects of inorganic nitrogen pollution in aquatic ecosystems: a global assessment. Environ Int.

[CR14] Chung KH, Lin YC, Lin AY (2018). The persistence and photostabilizing characteristics of benzotriazole and 5-methyl-1H-benzotriazole reduce the photochemical behavior of common photosensitizers and organic compounds in aqueous environments. Environ Sci Pollut Res.

[CR15] Cleuvers M (2003). Aquatic ecotoxicity of pharmaceuticals including the assessment of combination effects. Toxicol Lett.

[CR16] Cleuvers M (2004). Mixture toxicity of the anti-inflammatory drugs diclofenac, ibuprofen, naproxen, and acetylsalicylic acid. Ecotoxicol Environ Saf.

[CR17] Cleuvers M (2005). Initial risk assessment for three β-blockers found in the aquatic environment. Chemosphere.

[CR18] Comber S, Gardner M, Sörme P, Leverett D, Ellor B (2018). Active pharmaceutical ingredients entering the aquatic environment from wastewater treatment works: a cause for concern?. Sci Total Environ.

[CR19] Currens BJ, Hall AM, Brion GM, Fyar AE (2019). Use of acetaminophen and sucralose as con-analytes to differentiate sources of human excreta in surface waters. Water Res.

[CR20] Davis JR (1997). Revitalization of a north Texas river, as indicated by benthic macroinvertebrate communities. Hydrobiologia.

[CR21] De Oliveira LLD, Antunes SC, Gonçalves F, Rocha O, Nunes B (2016). Acute and chronic ecotoxicological effects of four pharmaceuticals drugs on cladoceran *Daphnia magna*. Drug Chem Toxicol.

[CR22] Drozdova J, Raclavska H, Skrobankova H (2015). A survey of heavy metals in municipal wastewater in combined sewer systems during wet and dry weather periods. Urban Water J.

[CR23] Environment Canada (2001) The state of municipal wastewater effluents in Canada (State of the Environment report). Indicators and Assessment Office, Environment Canada. Minister of Public Works and Government Services Canada, Ottawa, Ontario, Cat. No. En1-11/96E. Available from https://publications.gc.ca/site/eng/104183/publication.html. Accessed 21 Sept 2022

[CR24] Fent K, Weston AA, Caminada D (2006). Ecotoxicology of human pharmaceuticals. Aquat Toxicol.

[CR25] Gromaire MC, Garnaud S, Saad M, Chebbo G (2001). Contribution of different sources to the pollution of wet weather flows in combined sewers. Water Res.

[CR26] Isidori M, Lavorgna M, Nardelli A, Parrella A, Previtera L, Rubino M (2005). Ecotoxicity of naproxen and its phototransformation products. Sci Total Environ.

[CR27] Jenkins WR (1984) Acute toxicity of 1.6-dichloro-1,6-dideoxy-β−D-fructofuranosyl-4-chloro4-deoxy-α-D-galactopyranoside (TGS) to *Daphnia magna*. Aquatox Ltd

[CR28] Kaufmann A, Roth S, Ryser B, Widmer M (2002). Quantitative LC/MS-MS determination of sulfonamides and some other antibiotics in honey. J AOAC Int.

[CR29] Khalit WNAW, Tay KS (2017). The fate of sotalol in aqueous chlorination: kinetics, mechanisms and ecotoxicity assessment. Ecotox Environ Saf.

[CR30] Kwak K, Ji K, Kho Y, Kim P, Lee J, Ryu J, Choi K (2018). Chronic toxicity and endocrine disruption of naproxen in freshwater water fleas and fish, and steroidogenic alteration using H295R cell assay. Chemosphere.

[CR31] Kumar A, Batley GE, Nidumolu B, Hutchinson TH (2016). Derivation of water quality guidelines for priority pharmaceuticals. Environ Toxicol Chem.

[CR32] Launay MA, Dittmer U, Steinmetz H (2016). Organic micropollutants discharged by combined sewer overflows – characterisation of pollutant sources and stormwater-related processes. Water Res.

[CR33] Li J, Zhao H, Zhou Y, Xu S, Cai Z (2017). Determination of benzotriazoles and benzothiazoles in human urine by UHPLC-TQMS. J Chromatogr.

[CR34] Liu W, Xue J, Kannan K (2017). Occurrence of and exposure of benzothiazoles and benzotriazoles from textiles and infant clothing. Sci Total Environ.

[CR35] Lock S (2011) The quantitation and identification of artificial sweeteners in food and drink by liquid chromatography tandem mass spectrometry (LC-MS/MS) ABSciex Application Notes, Food & Environment

[CR36] MacRitchie E, Phipps K (2013) LC-MS/MS method for the rapid analysis of five artificial sweeteners using a core enhanced technology column. Thermofisher Application Note 20675

[CR37] Martínez-Pinilla E, Oñatibia-Astibia A, Franco R (2015). The relevance of theobromine for the beneficial effects of cocoa consumption. Front Pharmacol.

[CR38] MSDS (2015) Method safety data sheet version 1 for product 8X Laundry. Method Products Inc, San Francisco, California. Available at https://www.wholesalecleaningsupplies.com/image/data/msds/MTH01127_SDS.PDF. Accessed 8 Nov 2022

[CR39] Nagai T, Imai A, Matsushige K, Yokoi K, Fukushima T (2007). Dissolved iron and its speciation in a shallow eutrophic lake and its inflowing rivers. Water Res.

[CR40] NSW EPA (2022) NSW Environment Protection Authority, Environment Protection Licences 372. https://apps.epa.nsw.gov.au/prpoeoapp/Detail.aspx?instid=372&id=372&option=licence&searchrange=licence&range=POEO%20licence&prp=no&status=Issued and 378 https://apps.epa.nsw.gov.au/prpoeoapp/Detail.aspx?instid=378&id=378&option=licence&searchrange=licence&range=POEO%20licence&prp=no&status=Issued condition L7.1 (accessed 26 July 2022)

[CR41] Nunes B, Antunes S, Santos J, Martins L, Castro BB (2014). Toxic potential of paracetamol to freshwater organisms: a headache to environmental regulators?. Ecotox Environ Saf.

[CR42] Quijano JC, Zhu Z, Morales V, Landry BJ, Garcia MH (2017). Three-dimensional model to capture the fate and transport of combined sewer overflow discharges: a case study in the Chicago Area Waterway System. Sci Total Environ.

[CR43] Quinn B, Gagné F, Blaise C (2008). An investigation into the acute and chronic toxicity of eleven pharmaceuticals (and their solvents) found in wastewater effluent on the cnidarian, *Hydra attenuata*. Sci Total Environ.

[CR44] Quinn B, Gagné F, Blaise C (2009). Evaluation of the acute, chronic and teratogenic effects of a mixture of eleven pharmaceuticals on the cnidarian, *Hydra attenuata*. Sci Total Environ.

[CR45] Seeland A, Oetken M, Kiss A, Fries E, Oehlmann J (2012). Acute and chronic toxicity of benzotriazoles to aquatic organisms. Environ Sci Poll Res.

[CR46] Shi Z, Liu Y, Xiong Q, Cai W, Ying G (2019). Occurrence, toxicity and transformation of six typical benzotriazoles in the environment: a review. Sci Total Environ.

[CR47] Sieratowicz A, Kaiser D, Behr M, Oetken M, Oehlmann J (2011). Acute and chronic toxicity of four frequently used UV filter substances for *Desmodesmus subspicatus* and *Daphnia magna*. J Environ Sci Health A.

[CR48] Smyth DV (1986) Sucralose: determination of toxicity to the green alga *Selenastrum capricornutum*. ICI Brixham Laboratory; 1986

[CR49] Tjadraatmadja G, Diaper C (2006) Sources of critical contaminants in domestic wastewater – a literature review. Canberra, CSIRO: Water for a Healthy Country National Research Flagship Report. 10.4225/08/59b9805b74db1

[CR50] Tollefsen KE, Nizzetto L, Huggett DB (2012). Presence, fate and effects of the intense sweetener sucralose in the aquatic environment. Sci Total Environ.

[CR51] USEPA (2007) EPA Method 1694: Pharmaceuticals and personal care products in water, soil, sediment, and biosolids by HPLC/MS/MS. Office of Water, United States Environmental Protection Agency, Report No. EPA-821-R-08-002, Washington, DC, USA

[CR52] Vlasceanu AM, Olaru OT, Nitulescu GM, Baconi D (2015). Evaluation of the toxicity of nicotine and its metabolite cotinine on crustacean *Daphnia magna*. Toxicol Lett.

[CR53] Wang DC-Y, Chu W-L, Kok Y-Y (2015). Assessment of paracetamol (acetaminophen) toxicity in microalgae. Pol J Environ Stud.

[CR54] White D, Lapworth DJ, Civil W, Williams P (2019). Tracking changes in the occurrence and source of pharmaceuticals within the River Thames, UK; from source to sea. Environ Pollut.

[CR55] Williams TD (1986). Sucralose: determination of effects on the survival and reproduction of *Daphnia magna*.

[CR56] Yamamoto H, Nakamura Y, Moriguchi S, Nakamura Y, Honda Y, Tamura I, Hirata Y, Hayashi A, Sekizawa J (2009). Persistence and partitioning of eight selected pharmaceuticals in the aquatic environment: laboratory photolysis, biodegradation, and sorption experiments. Water Res.

[CR57] Zhang J, Zhang X, Wu L, Wang T, Zhao J, Zhang Y, Men Z, Mao H (2018). Occurrence of benzothiazole and its derivates in tire wear, road dust, and roadside soil. Chemosphere.

[CR58] Zhang Z, Ren N, Li Y, Kunisue T, Gao D, Kannan K (2011). Determination of benzotriazole and benzophenone UV filters in sediment and sewage sludge. Environ Sci Technol.

[CR59] Zhao X, Zhang ZF, Xu L, Liu LY, Song WW, Zhu FJ, Li YF, Ma WL (2017). Occurrence and fate of benzotriazoles UV filters in a typical residential wastewater treatment plant in Harbin, China. Environ Pollut.

