# Supplementary material for: Tracking contaminants of concern in wet-weather sanitary sewer overflows
Source: Environ Sci Pollut Res Int. 2023 Aug 15;30(43):96763–81. doi: 10.1007/s11356-023-29152-x (PMC10495504; doi:10.1007/s11356-023-29152-x)
Supplement: Supplementary file 1 — (DOCX 2282 kb) [file 11356_2023_29152_MOESM1_ESM.docx]

**Supporting Information**

**Tracking contaminants of concern in wet-weather sanitary sewer overflows**

**Colin H. Besley^1*^, Graeme E. Batley^2^, and Michele Cassidy^3^**

^1^ Laboratory Services, Sydney Water, 51 Hermitage Road, West Ryde, NSW, 2114, Australia

^2^ CSIRO Environment, Locked Bag 2007, Kirrawee, NSW 2232, Australia

^3^ Wastewater Product, Sydney Water, 1 Smith Street, Parramatta, NSW, 2150, Australia

* corresponding author: colin.besley@sydneywater.com.au

**Table S1** Modelled overflow volume and spill frequency at ERSs

| **Study location and (Asset Generated Number)** | **Modelled volume in 10 years (ML)** | **Modelled overflow spill frequency in 10 years** | **Carrier pipe diameter (mm)** |
| --- | --- | --- | --- |
| Vineyard (8723124) | 2117 | 64 | 1830 and 2100 |
| Darling Mills (1395315) | 607 | 52 | 750 |
| Buffalo (1283126) | 568 | 134 | 750 |
| Gymea (1270912) | 206 | 15 | 1200 |

**Table S2.** Instrumental conditions for in-house Method TC0063 for benzotriazoles

Column: Water Acquity UHPLC BEH C18 Column 2.1 mm x 100 mm, 1.7 µm or equivalent

Mobile phase A: 0.1% formic acid in ultrapure (Type 1) water;

Mobile phase B: 90% acetonitrile in ultrapure (Type 1) water

Oven temperature: 40°C

Injection volume: 25 µL

Analysis (Run) Time: 8 minutes

Rinses

R0- Solvent A

R1- Weak wash 80% ultrapure (Type 1) water + 20% methanol

R2- Strong wash 50% acetonitrile + 50% ultrapure (Type 1) water

**LC gradient Table**

| **Time (min)** | **Module** | **Flow (mL/min)** | **Solvent A %** | **Solvent B %** |
| --- | --- | --- | --- | --- |
| 0 | Pumps | 0.3 | 90 | 10 |
| 2.0 | Pumps | 0.3 | 90 | 10 |
| 5.0 | Pumps | 0.3 | 0 | 100 |
| 6.0 | Pumps | 0.3 | 0 | 100 |
| 6.1 | Pumps | 0.3 | 90 | 10 |
| 8.0 | System Controller | Stop |  |  |

MSMS Conditions:

| **Parameter** |  |  |
| --- | --- | --- |
| Polarity |  | Positive |
| CUR |  | 35 |
| CAD |  | Medium |
| TEM |  | 450 |
| GS1 |  | 50 |
| GS2 |  | 50 |
| ihe |  | ON |
| IS |  | 5500 |

Multiple reaction monitoring detection window - 60 s

Target scan time: Experiment 1 (Positive) 1.0 s

**Table S3.** Scheduled multiple reaction monitoring (MRM) conditions Sciex 6500 for in-house Method TC0063 for benzotriazoles

| **Analyte** | **Q1 Mass** | **Q3 Mass** | **Time (mins)** | **Dwell weight** | **DP** | **EP** | **CE** | **CXP** |
| --- | --- | --- | --- | --- | --- | --- | --- | --- |
| 1H-Benzotriazole-1 | 119.0 | 65.0 | 2.00 | 5 | 15 | 10 | 30 | 5 |
| 1H-Benzotriazole -2 | 119.0 | 92.0 | 2.00 | 5 | 140 | 10 | 22 | 5 |
| 1H-Benzotriazole -3 | 119.0 | 39.0 | 2.00 | 5 | 140 | 10 | 45 | 5 |
| 4-Methyl-benzotriazole -1 | 133.5 | 76.5 | 2.08 | 5 | 130 | 10 | 30 | 10 |
| 4-Methyl-benzotriazole -2 | 133.5 | 106.1 | 2.08 | 5 | 140 | 10 | 22.0 | 10 |
| 5-Methyl-benzotriazole -1 | 134.2 | 77.0 | 2.08 | 3 | 245 | 10 | 40 | 10 |
| 5-Methyl-benzotriazole -2 | 134.2 | 106.0 | 2.08 | 3 | 180 | 10 | 23 | 5 |

DP = Declustering Potential; EP = Entrance Potential; CE = Collision Energy; CXP = Collision Cell Exit Potential

**Table S4.** Instrumental conditions for in-house Method TC0065 for pharmaceuticals and personal-care products and other organics

Column: Water Acquity UHPLC BEH C18 Column 2.1 mm x 100 mm, 1.7 µm or equivalent

Mobile Phase: A - 1 mM ammonium fluoride

Mobile phase: B - methanol

Oven temperature: 40°C

Injection volume: 20 µL

Analysis (Run) Time: 15 minutes

Rinses

R0- 1 mM ammonium fluoride (mobile phase A)

R1- Weak wash 95% ultrapure (Type 1) water 5% acetonitrile + 1% formic acid

R2- Strong wash 25% acetonitrile +25% methanol +50% ultrapure (Type 1) water + 1% formic acid

LC gradient Table

| **Time (min)** | **Module** | **Flow (mL/min)** | **Solvent A %** | **Solvent B %** |
| --- | --- | --- | --- | --- |
| 0 | Pumps | 0.3 | 80 | 20 |
| 0.5 | Pumps | 0.3 | 80 | 20 |
| 2.0 | Pumps | 0.3 | 70 | 30 |
| 5.0 | Pumps | 0.3 | 40 | 60 |
| 7.0 | Pumps | 0.3 | 0 | 100 |
| 11.50 | Pumps | 0.3 | 0 | 100 |
| 11.55 | Pumps | 0.3 | 80 | 20 |
| 13.50 | System Controller | Stop |  |  |

MSMS Conditions

| **Parameter** |  |  |
| --- | --- | --- |
| Polarity | Negative | Positive |
| CUR | 30 | 30 |
| CAD | Medium | Medium |
| TEM | 400 | 400 |
| GS1 | 60 | 60 |
| GS2 | 50 | 50 |
| ihe | ON | ON |
| IS | -4500 | 5500 |

Multiple reaction monitoring detection window - 60 s

Target scan time:

Experiment 1 (Negative) 0.35 s

Experiment 2 (Positive) 0.35 s

**Table S5.** Scheduled multiple reaction monitoring (MRM) conditions Sciex 6500 for in-house Method TC0065 for pharmaceuticals and personal-care products and other organics

| **Analyte** | **Q1 Mass** | **Q3 Mass** | **Time (mins)** | **Dwell weight** | **DP** | **EP** | **CE** | **CXP** |
| --- | --- | --- | --- | --- | --- | --- | --- | --- |
| Metformin-1 | 130.4 | 60.0 | 1.3 | 1 | 40 | 10 | 18 | 10 |
| Metformin-2 | 130.4 | 71.0 | 1.3 | 1 | 40 | 10 | 22 | 5 |
| Acetaminophen -1 | 152.1 | 110.1 | 3.1 | 1 | 80 | 10 | 20 | 5 |
| Acetaminophen-2 | 152.1 | 65.0 | 3.1 | 1 | 80 | 10 | 35 | 5 |
| Atenolol-1 | 267.2 | 145.2 | 3.6 | 1 | 80 | 10 | 35 | 4 |
| Atenolol-2 | 267.2 | 190.2 | 3.6 | 1 | 80 | 10 | 20 | 4 |
| Theobromine-1 | 181.0 | 137.6 | 3.2 | 1 | 140 | 10 | 27 | 5 |
| Theobromine-2 | 181.0 | 108.1 | 3.2 | 1 | 140 | 10 | 27 | 10 |
| Sotalol-1 | 272.6 | 175.8 | 2.6 | 1 | 60 | 10 | 30 | 5 |
| Sotalol-2 | 276.2 | 254.6 | 2.6 | 1 | 60 | 10 | 20 | 5 |
| Sulfapyridine-1 | 250.6 | 156.0 | 4.6 | 1 | 140 | 10 | 26 | 5 |
| Sulfapyridine-2 | 250.6 | 180.0 | 4.6 | 1 | 140 | 10 | 30 | 5 |
| Cotinine-1 | 177.1 | 98.3 | 5.7 | 1 | 40 | 10 | 30 | 5 |
| Cotinine-2 | 177.1 | 146.0 | 5.7 | 1 | 40 | 10 | 23 | 10 |
| Sucralose-1 | 414.1 | 199.0 | 5.8 | 1 | 60 | 10 | 20 | 5 |
| Sucralose-2 | 414.1 | 200.9 | 5.8 | 1 | 60 | 10 | 20 | 15 |
| Benzophenone3-1 | 229.1 | 105.0 | 8.3 | 1 | 20 | 10 | 20 | 5 |
| Benzophenone3-2 | 229.1 | 151.1 | 8.3 | 1 | 20 | 10 | 24 | 10 |
| Disodium distyrylbiphenyl disulfonate -1 | 536.2 | 455.1 | 7.1 | 1 | 20 | 10 | 30 | 5 |
| Disodium distyrylbiphenyl disulfonate -2 | 536.2 | 349.2 | 7.1 | 1 | 20 | 10 | 40 | 5 |
| p-Toluenesulfonamide -1 | 170.1 | 78.9 | 5.6 | 1 | -40 | -10 | -30 | -10 |
| p-Toluenesulfonamide -2 | 170.1 | 63.9 | 5..6 | 1 | -40 | -10 | -60 | -5 |
| Hydrochlorothiazide -1 | 296.1 | 268.9 | 2.9 | 1 | -20 | -10 | -25 | -10 |
| Hydrochlorothiazide -2 | 296.1 | 204.9 | 2.9 | 1 | -20 | -10 | -30 | -8 |
| Naproxen-1 | 229.0 | 170.1 | 7.2 | 1 | -90 | -10 | -22 | -15 |
| Naproxen-2 | 229.0 | 169.0 | 7.2 | 1 | -90 | -10 | -44 | -15 |
| Diclofenac-1 | 294.0 | 250.0 | 7.8 | 1 | -45 | -10 | -16 | -17 |
| Diclofenac-2 | 296.0 | 252.0 | 7.8 | 1 | -45 | -10 | -18 | -15 |
| Ibuprofen-1 | 205.1 | 161.2 | 8.3 | 1 | -60 | -10 | -10 | -16 |
| Ibuprofen-2 | 205.1 | 159.0 | 8.3 | 1 | -60 | -10 | -10 | -16 |

DP = Declustering Potential; EP = Entrance Potential; CE = Collision Energy; CXP = Collision Cell Exit Potential

**Table S6** Percentile distribution of contaminant concentrations (µg/L) in receiving waters downstream of ERSs when a spill was occurring between October 2018 and February 2020

| **Contaminant** | **Acetaminophen** | | | | | **Cotinine** | | | | | **FB351** | | | | | **Ibuprofen** | | | | |
| --- | --- | --- | --- | --- | --- | --- | --- | --- | --- | --- | --- | --- | --- | --- | --- | --- | --- | --- | --- | --- |
|  | **Vineyard** | **Buffalo** | **Darling Mills** | **Gymea 1** | **Gymea 2** | **Vineyard** | **Buffalo** | **Darling Mills** | **Gymea 1** | **Gymea 2** | **Vineyard** | **Buffalo** | **Darling Mills** | **Gymea 1** | **Gymea 2** | **Vineyard** | **Buffalo** | **Darling Mills** | **Gymea 1** | **Gymea 2** |
| Minimum | <1 | <1 | <1 | <1 | <1 | <1 | <1 | <1 | <1 | <1 | . | <1 | . | <1 | <1 | <1 | <1 | <1 | <1 | <1 |
| Median | 7 | <1 | <1 | <1 | <1 | <1 | <1 | <1 | <1 | <1 | . | <1 | . | <1 | <1 | <1 | <1 | <1 | <1 | <1 |
| 75th percentile | 23 | <1 | <1 | <1 | <1 | <1 | <1 | <1 | <1 | <1 | . | <1 | . | <1 | <1 | <1 | <1 | <1 | <1 | <1 |
| 90th percentile | 33 | <1 | <1 | <1 | <1 | <1 | <1 | <1 | <1 | <1 | . | <1 | . | <1 | <1 | <1 | <1 | <1 | <1 | <1 |
| Maximum | 98 | 1 | <1 | <1 | <1 | <1 | <1 | <1 | <1 | <1 | . | <1 | . | <1 | <1 | 3 | <1 | <1 | <1 | <1 |
| **Contaminant** | **Metformin** | | | | | **Naproxen** | | | | | **Sucralose** | | | | | **Sulfapyridine** | | | | |
|  | **Vineyard** | **Buffalo** | **Darling Mills** | **Gymea 1** | **Gymea 2** | **Vineyard** | **Buffalo** | **Darling Mills** | **Gymea 1** | **Gymea 2** | **Vineyard** | **Buffalo** | **Darling Mills** | **Gymea 1** | **Gymea 2** | **Vineyard** | **Buffalo** | **Darling Mills** | **Gymea 1** | **Gymea 2** |
| Minimum | 2 | <1 | <1 | <1 | <1 | <1 | <1 | <1 | <1 | <1 | <1 | <1 | <1 | <1 | <1 | <1 | <1 | <1 | <1 | <1 |
| Median | 8 | <1 | <1 | <1 | <1 | <1 | <1 | <1 | <1 | <1 | 1 | <1 | <1 | <1 | <1 | <1 | <1 | <1 | <1 | <1 |
| 75th percentile | 9 | <1 | <1 | <1 | <1 | <1 | <1 | <1 | <1 | <1 | 2 | <1 | <1 | <1 | <1 | <1 | <1 | <1 | <1 | <1 |
| 90th percentile | 11 | <1 | <1 | <1 | <1 | <1 | <1 | <1 | <1 | <1 | 3 | <1 | <1 | <1 | <1 | <1 | <1 | <1 | <1 | <1 |
| Maximum | 11 | <1 | <1 | <1 | <1 | <1 | <1 | <1 | <1 | <1 | 7 | <1 | <1 | <1 | <1 | <1 | <1 | <1 | <1 | <1 |
| **Contaminant** | **Theobromine** | | | | | **1H-benzotriazole** | | | | | **4-Methyl-1H-benzotriazole** | | | | | **5-Methyl-1H-benzotriazole** | | | | |
|  | **Vineyard** | **Buffalo** | **Darling Mills** | **Gymea 1** | **Gymea 2** | **Vineyard** | **Buffalo** | **Darling Mills** | **Gymea 1** | **Gymea 2** | **Vineyard** | **Buffalo** | **Darling Mills** | **Gymea 1** | **Gymea 2** | **Vineyard** | **Buffalo** | **Darling Mills** | **Gymea 1** | **Gymea 2** |
| Minimum | <1 | <1 | <1 | <1 | <1 | <0.25 | <0.25 | <0.25 | <0.25 | <0.25 | <0.25 | <0.25 | <0.25 | <0.25 | <0.25 | <0.25 | <0.25 | <0.25 | <0.25 | <0.25 |
| Median | 2 | <1 | <1 | <1 | <1 | <0.25 | <0.25 | <0.25 | <0.25 | <0.25 | <0.25 | <0.25 | <0.25 | <0.25 | <0.25 | <0.25 | <0.25 | <0.25 | <0.25 | <0.25 |
| 75th percentile | 3 | <1 | <1 | <1 | <1 | 0.4 | <0.25 | <0.25 | <0.25 | <0.25 | <0.25 | <0.25 | <0.25 | <0.25 | <0.25 | <0.25 | <0.25 | <0.25 | <0.25 | <0.25 |
| 90th percentile | 4 | <1 | <1 | <1 | <1 | 0.5 | 0.3 | <0.25 | 0.5 | <0.25 | <0.25 | <0.25 | <0.25 | <0.25 | <0.25 | <0.25 | <0.25 | <0.25 | <0.25 | <0.25 |
| Maximum | 10 | <1 | <1 | <1 | <1 | 0.7 | 0.5 | <0.25 | 3 | <0.25 | 0.4 | 0.8 | <0.25 | <0.25 | <0.25 | 0.8 | 0.8 | <0.25 | <0.25 | <0.25 |

Detection limits of <0.25 µg/L for three forms of benzotriazole and <1 µg/L for other nine contaminants with substituted values of 0.125 and 0.5 applied in distribution calculation;

Vineyard: 39 samples were collected with standards not to hand for laboratory analysis of FB351 and for 30 samples of metformin;

Buffalo: 25 samples were collected and analysed for all 12 contaminants;

Darling Mills: 20 samples were collected with standards not to hand for laboratory analysis of FB351 and for 10 samples of metformin;

Gymea 1: 27 samples were collected and analysed for 11 contaminants while 9 samples were not analysed for FB351;

Gymea 2: 41 samples were collected and analysed for 11 contaminants while 15 samples were not analysed for FB351.

**Table S7** Percent detection across autosampler collection events at five receiving water locations

|  | **Vineyard** | **Buffalo** | **Darling Mills** | **Gymea 1** | **Gymea 2** |
| --- | --- | --- | --- | --- | --- |
| **Events** | **8** | **5** | **4** | **7** | **7** |
| 1H-Benzotriazole | 62% | 40% | nd | 43% | nd |
| 4-Methyl-1H-benzotriazole | 12% | 20% | nd | nd | nd |
| 5-Methyl-1H-benzotriazole | 12% | 20% | nd | nd | nd |
| Acetaminophen | 87% | 40% | nd | nd | nd |
| Ibuprofen | 12% | nd | nd | nd | nd |
| Metformin | 100%^a^ | nd | nd | nd | nd |
| Sucralose | 75% | nd | nd | nd | nd |
| Theobromine | 100% | nd | nd | nd | nd |

nd = not detected; ^a^laboratory standards only to hand for last two collection events, as such percentage calculation based on two events


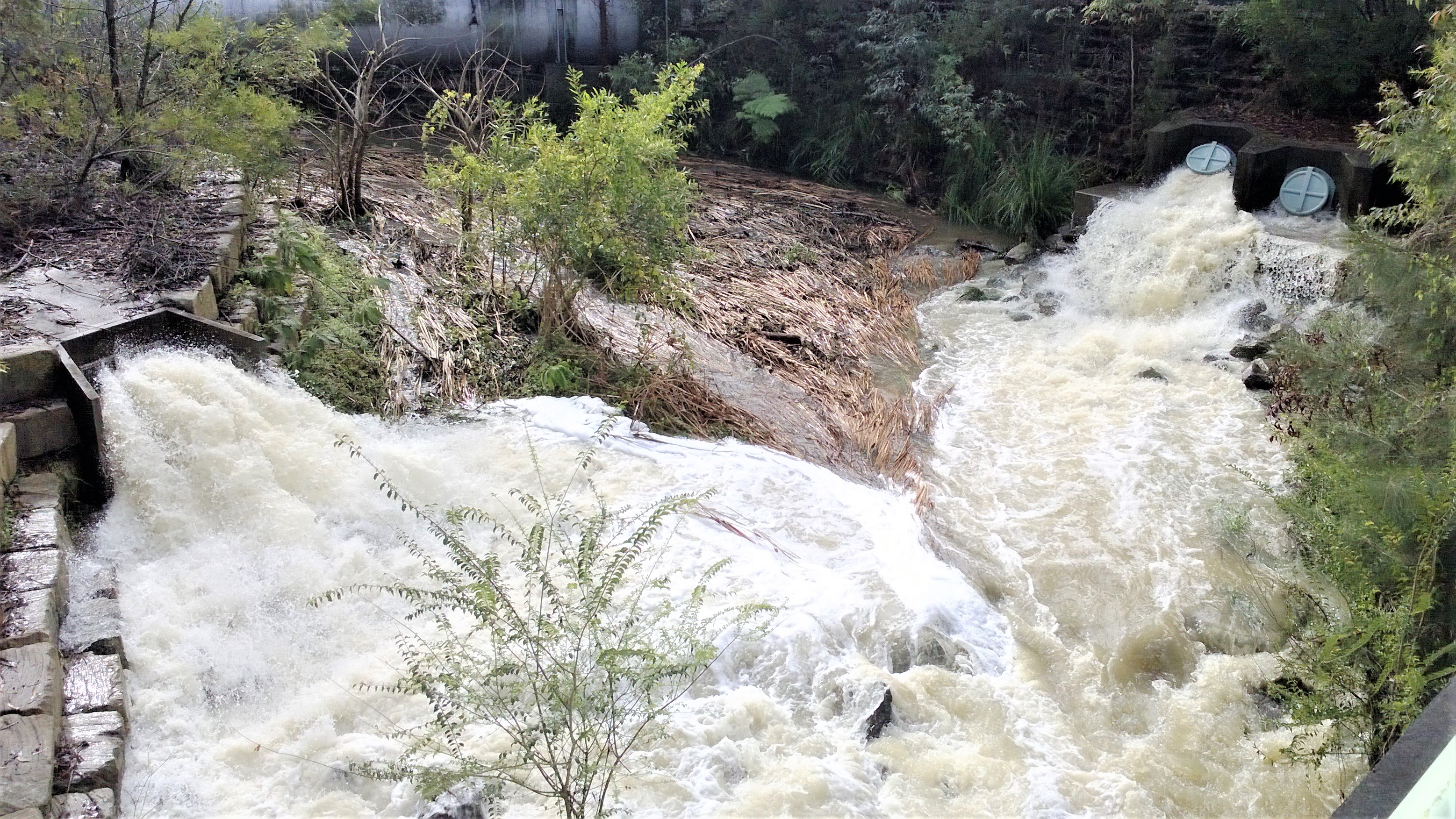


**Figure S1.** Three ERSs at the Vineyard location spilling to Vineyard Creek. Flows from the siphonic discharge structure are on the left-hand side of the image.
